# Refining the concept of GFAP toxicity in Alexander disease

**DOI:** 10.1186/s11689-019-9290-0

**Published:** 2019-12-16

**Authors:** Albee Messing

**Affiliations:** Waisman Center and Department of Comparative Biosciences, University of Wisconsin-Madison, 1500 Highland Ave, Rm. 713B, Madison, WI 53705 USA

**Keywords:** GFAP, Astrocyte, Antisense oligonucleotides

## Abstract

**Background:**

Alexander disease is caused by dominantly acting mutations in glial fibrillary acidic protein (GFAP), the major intermediate filament of astrocytes in the central nervous system.

**Main body:**

In addition to the sequence variants that represent the origin of disease, GFAP accumulation also takes place, together leading to a gain-of-function that has sometimes been referred to as “GFAP toxicity.” Whether the nature of GFAP toxicity in patients, who have mixtures of both mutant and normal protein, is the same as that produced by simple GFAP excess, is not yet clear.

**Conclusion:**

The implications of these questions for the design of effective treatments are discussed.

## Background

Alexander disease, once a minor disorder known primarily to neuropathologists and the small number of neurologists who followed the leukodystrophies, has now gained increased attention as the first well-documented example of a primary disease of astrocytes. Since the first case report by W. Stewart Alexander [1], its recognition as a distinct entity evolved over time (prolonged due to the rarity of the disease). By the late 1960s, the condition had been recognized in both children and adults, although its origin remained a mystery. A genetic basis was presumed, but not identified. The breakthrough came from the world of mouse genetics. In studies originally designed to study the role of intermediate filaments in reactive astrocytosis (or “gliosis”), transgenic mouse models were designed to force over-expression of the major astrocyte intermediate filament, GFAP. The surprising result was that astrocytes in these mice formed the hallmark protein aggregates of Alexander disease—Rosenthal fibers [2]. Mice engineered to have the highest levels of GFAP died within weeks after birth. Although these mice did not have a leukodystrophy, the link between GFAP and Rosenthal fibers provided sufficient rationale to pursue *GFAP* as a candidate gene for the disease [3]. We and others quickly found that nearly all Alexander disease patients carried heterozygous missense mutations in the coding region of *GFAP*, and that such mutations could account for all forms of the disease [4, 5].

The dominant nature of the GFAP variants, coupled with the minimal phenotype associated with complete GFAP deficiency as illustrated in mouse knockouts, supports the hypothesis that Alexander disease is a gain-of-function disease [6, 7]. Indeed, no null variants have ever been found in human patients. That the hallmark aggregate, Rosenthal fibers, could be induced by forced over-expression, and that this by itself could be lethal (in the mouse), also led to the idea of GFAP “toxicity” (due to GFAP excess) as a unifying hypothesis to explain how mutations cause disease. However, focusing only on GFAP excess is a mistake. The goal of this brief review is to stress the broader concept that the initiating event in disease pathogenesis must be mutant GFAP, and that this occurs prior to any change in levels. Any subsequent rise in total GFAP would then act to exacerbate the disease process.

## Main text

**Fig. 1:**
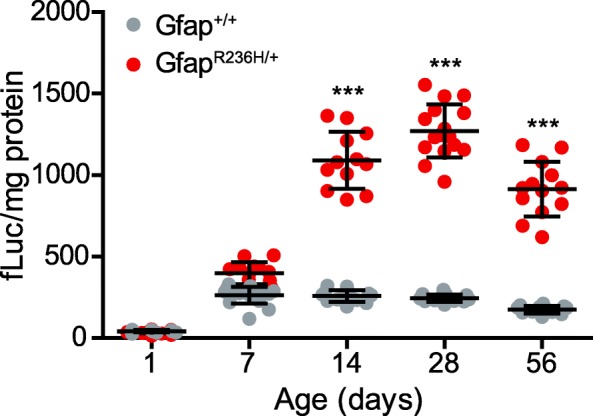
Increase in activity of the *Gfap* promoter during early postnatal development of a mouse model of Alexander disease. Promoter activity (monitored through expression of a *Gfap-*luciferase reporter) in mutants rises above that in wild-type mice between postnatal days 7 to 14 and remains elevated through at least 8 weeks of age. Figure used with permission [11]

GFAP levels are indeed elevated in Alexander disease, and one important question is why? Given the significant tissue damage that often exists, along with the expected reactive response by astrocytes, it is no surprise that GFAP levels rise. In a series of six patients, Walker et al. [8] showed increased levels of protein that at least roughly corresponded to the severity of disease (as defined by age of onset). Increased levels of GFAP mRNA had previously been documented for two patients by Hageman et al. [9], and so one can assume that increased synthesis is at least one mechanisms contributing to the overall change in levels. Mouse models engineered to carry a disease-associated variant in their endogenous *Gfap* gene also display increased levels of both mRNA and protein [10]. Using luciferase reporter lines of mice that serve as indirect monitors of the murine *Gfap* promoter, Jany et al. [11] found that mutant mice dramatically increase promoter activity during the second postnatal week, and in the absence of any significant pathology (Fig. 1). Hence, we believe this change in GFAP expression reflects an early and spontaneous alteration of astrocyte function, equivalent to activation of other downstream stress pathways, which in this particular instance unfortunately upregulates expression of the very protein that is causative for disease. A recent study using a transgenic mouse expressing the human R239H variant suggests that abnormal calcium signaling may be a key factor contributing to the upregulation of GFAP [12].

In theory, impaired degradation could also contribute to the accumulation of GFAP protein, but the evidence for this is less certain. Early results suggested that mutant GFAPs interfered with function of the proteasome [13]. Furthermore, the small heat shock protein, αB-crystallin, which had previously been shown to be protective in mouse models of the disease [14], could relieve the block on the proteasome through binding to small oligomers of GFAP [15]. However, other evidence indicated that autophagy might be increased [16], so that the net effect on overall degradation rate remained unclear. Recently, we investigated the turnover rate of GFAP directly in mouse models, by introducing amino acids containing heavy nitrogen via the diet and following the conversion of the total GFAP population from the light to heavy forms of nitrogen using mass spectrometry [17]. To our surprise, the turnover rate of GFAP in the mutant mice was roughly twice as fast as in control mice, indicating that degradation must have increased, at least in the adult animals that were the focus of this study.

**Fig. 2:**
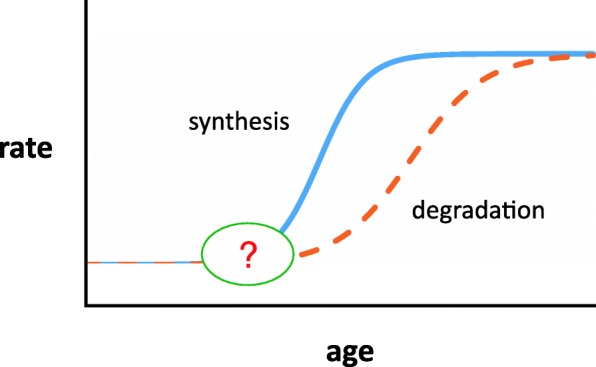
Proposed model for changes in rates of synthesis and degradation of GFAP caused by the presence of mutant protein. Initially rates of synthesis and degradation are equal, with stable levels of protein. A change occurs which increases synthesis, but there is a lag period before degradation increases, during which the imbalance results in increased levels of protein. Eventually, synthesis and degradation reach a new equilibrium, but maintaining a higher level of total GFAP. Figure used with permission [18]

The simplest explanation of these results is that an early event in pathogenesis is an increase in synthesis, followed by an undetermined lag period after which degradation also increases (Fig. 2). If degradation had increased immediately to match the change in synthesis, no change in protein levels would occur. It is the lag in the compensatory response that allows protein levels to rise, eventually reaching a new but higher equilibrium. What initiates the change in synthesis is not yet known, but presumably reflects activation of one or more cellular stress pathways by the initial production of even small amounts of mutant protein. One way or another, GFAP levels are elevated in Alexander disease. This phenomenon begins in the astrocyte, but is also evident in the cerebrospinal fluid (CSF) of most patients and even in blood of some [19]. The release of detectable levels of GFAP into body fluids that are more accessible for biopsy is a feature that may prove useful for following the response to experimental therapies.

The question of whether GFAP elevation by itself causes all of the downstream effects (i.e., microglial activation, neuronal and oligodendroglial dysfunction) is unresolved. Clearly some aspects of the disease are replicated simply by increased levels of normal protein, such as formation of Rosenthal fibers and activation of multiple stress response pathways. However, no patients have yet been identified with disease caused by excess GFAP of normal sequence, such as might be produced by GFAP duplications [20]. We believe, but do not yet have proof, that the threshold for toxicity is lower when mutant protein is involved. It is interesting that, in the mouse at least, GFAP is not uniformly elevated in all regions of the CNS. Indeed, the cervical spinal cord, a site that is consistently affected in human Alexander disease, shows only modest increase in *Gfap* mRNA and even a decrease in the level of protein [11]. This occurs despite the clear presence of Rosenthal fibers in this location. Whether the same anomaly occurs in human spinal cord has not yet been investigated.

Recently, two publications have appeared that report the generation and characterization of induced pluripotent stem cells (iPSC) derived from patients with Alexander disease [21, 22]. In both cases, astrocytes differentiated from these iPS cells form Rosenthal-like fibers and acquire many distinct abnormalities compared with isogenic controls in which the GFAP variant was corrected to the normal sequence. Although Li et al. [21] do not comment on whether GFAP levels differed between the mutant and control cell lines, Jones et al. [22] assert many phenotypic changes that take place in the absence of evident change in GFAP.

In the disease setting, where patients are heterozygous, the GFAP pool consists of both normal and mutant protein, and it would be very useful to be able to distinguish one from the other. However, antibodies capable of distinguishing the two (which usually differ in only a single amino acid) exist for just one variant—the Arg416Trp mutation. In this case, Perng et al. [23] showed that both proteins were present within the Rosenthal fibers of a human patient carrying this mutation. Using a cell-free assay designed to study assembly of individual monomers into mature 10-nm intermediate filaments, they could show that having just 25% mutant protein was sufficient to cause aggregation.

In another patient, reported by Flint et al. [24], an unusual splice site mutation resulted in an in-frame deletion of exon 4, predicting synthesis of a protein missing 54 amino acids (207–260 of the normal 432) in the rod domain. For this patient, brain mRNA was available for analysis, which revealed that only 8% of the GFAP mRNA derived from the mutant allele. Using a cell culture model for studying filament assembly, they found that the mutant protein could disrupt polymerization even at very low levels, or 2.5% of the total.

Since so little is understood about the normal functions of GFAP, it is difficult to discuss in precise terms exactly how Alexander disease-associated mutations might cause gain or loss of any particular functions. The arguments reviewed above support the idea that increasing total levels replicate some key aspects of disease, and if mutant proteins acquire new and toxic properties, this qualifies as a different type of gain-of-function. Dominant negative mutations, which are genetically dominant but produce phenotypes that often resemble the complete deficiency state modeled by mouse knockouts, are well-known causes of other disorders. But Alexander disease bears little resemblance to the minimal phenotype observed in mouse knockouts of GFAP. With the present state of knowledge about GFAP and Alexander disease, we must accept the possibility that disease reflects a combination of both gain and loss of different functions, although we would argue that ultimately it is the gain-of-function that dominates.

## Conclusion

What implications do these findings have for design of potential treatments? Several approaches have been proposed in the past, targeting different downstream effects of mutant protein [25], but the most straightforward idea is that of reducing or eliminating production of the protein that initiates the disease process—GFAP. The number of known disease-causing variants already exceeds 100, a seemingly insurmountable number for a strategy of allele-specific suppression. At present, the most feasible means for reducing GFAP is generalized suppression, involving reduction of both mutant and normal protein. The rationale for GFAP suppression remains the same whether one starts from a baseline of apparently normal levels (with some being mutant) or the elevated levels seen in most patients or regions. Previous attempts to identify suppressors of GFAP expression through screens of known drugs or compounds suffered from modest or inconsistent effects (clomipramine— [26]), lack of in vivo data (curcumin— [27]), or unacceptable side effects (lithium— [28]).

A dramatic advance on the therapeutic front is our recently reported finding that antisense oligonucleotides (ASOs) are a remarkably effective means for suppressing GFAP expression, and can even reverse established pathology [29]. The effects of single intracerebroventricular injections of such ASOs become manifest within weeks after injection and persist for several months. Rosenthal fibers disappear, and several downstream markers of activated astrocytes and/or microglia return close to normal levels.

The degree to which astrocytes are completely normalized by ASO suppression remains to be seen. Nevertheless, these findings have generated considerable interest in the clinical community and offer the first real promise of a therapeutic worth testing in a formal clinical trial. ASO approaches for neurological diseases are already approved or in advanced stages of clinical development for other conditions, such as spinal muscular atrophy, Huntington’s disease, and amyotrophic lateral sclerosis [30]. For Alexander disease, application of the ASO approach to treatment will require better understanding of how closely the GFAP levels in CSF and blood reflect those in the brain and spinal cord, so that each individual’s response to treatment can be assessed in the least invasive way possible. In addition, it is important to recognize that human patients typically have more extensive pathology than any of the animal models to date, and the degree of rescue that is achievable in the clinical setting will only be learned through experience, and may require the adoption of secondary forms of treatment that complement the reduction or elimination of toxic GFAP.

## Data Availability

Not applicable

## Abbreviations

ASO: Antisense oligonucleotide
CSF: Cerebrospinal fluid
GFAP: Glial fibrillary acidic protein
iPSC: Induced pluripotent stem cells
